# Inferring Indel Parameters using a Simulation-based Approach

**DOI:** 10.1093/gbe/evv212

**Published:** 2015-11-03

**Authors:** Eli Levy Karin, Avigayel Rabin, Haim Ashkenazy, Dafna Shkedy, Oren Avram, Reed A. Cartwright, Tal Pupko

**Affiliations:** ^1^Department of Cell Research and Immunology, George S. Wise Faculty of Life Sciences, Tel-Aviv University, Tel-Aviv, Israel; ^2^The Blavatnik School of Computer Science, Tel-Aviv University, Tel-Aviv, Israel; ^3^The Biodesign Institute, Arizona State University, Tempe; ^4^School of Life Sciences, Arizona State University, Tempe

**Keywords:** simulations, phylogeny, indels, alignments, Mahalanobis distance

## Abstract

In this study, we present a novel methodology to infer indel parameters from multiple sequence alignments (MSAs) based on simulations. Our algorithm searches for the set of evolutionary parameters describing indel dynamics which best fits a given input MSA. In each step of the search, we use parametric bootstraps and the Mahalanobis distance to estimate how well a proposed set of parameters fits input data. Using simulations, we demonstrate that our methodology can accurately infer the indel parameters for a large variety of plausible settings. Moreover, using our methodology, we show that indel parameters substantially vary between three genomic data sets: Mammals, bacteria, and retroviruses. Finally, we demonstrate how our methodology can be used to simulate MSAs based on indel parameters inferred from real data sets.

## Introduction

A large body of research is dedicated to understanding how the evolutionary process varies within groups of orthologs, among sites within a gene, between populations, and among diverged species. Evolutionary models aiming to describe these dynamics must account for base pair substitutions as well as insertion and deletion (indel) events.

Great progress has been made in developing rich and accurate substitution models that account for many features of the evolutionary process. Such features include accounting for differences between rates of transitions and transversions as implemented, for example, in the HKY85 model (Hasegawa et al. 1985), accounting for among-site rate variation by using a Gamma distribution (reviewed in Yang 1996; Pupko and Mayrose 2010), considering heterotachy (Whelan et al. 2011), lifting the assumption of stationarity (Barry and Hartigan 1987; Lockhart et al. 1994), and accounting for dependencies among sites (Yang 1995; Robinson et al. 2003; Siepel and Haussler 2004; Rodrigue et al. 2005; Stern and Pupko 2006; Suzuki et al. 2009; Berard and Gueguen 2012).

Following the pioneering TKF (Thorne, Kishino, and Felsenstein) models (Thorne et al. 1991, 1992), several models to describe indel dynamics have been proposed: From statistical alignment algorithms (Lunter et al. 2003), through the development of the long indel model (Miklos et al. 2004), to the use of the Poisson distribution to describe a fixed instantaneous rate of indels (Cartwright 2005). The length distribution of indels has been studied in various biological data sets and was proposed to follow a Zipf (power law) distribution (Benner et al. 1993; Gu and Li 1995; Zhang and Gerstein 2003; Chang and Benner 2004; Zhang et al. 2010). This distribution describes an inverse relation between the length of an indel (k) and its probability: $\text{Pr}\left(\text{k}\right)=\frac{{\text{k}}^{-\text{a}}}{\text{ζ}\left(\text{a}\right)}$, where *a* > 1 is the slope parameter of the distribution, and $\zeta \left(a\right)=\overset{\infty }{\underset{n=1}{\sum }}n^{-a}$ is the Riemann zeta function.

When utilizing substitution models, free parameters are usually inferred from data. For example, many applications rely on the inference of the parameter of the gamma distribution used to model among-site rate variation (Buckley et al. 2001; Susko et al. 2003; Pond and Frost 2005; Abhiman et al. 2006; Rubinstein et al. 2011). Another example is the evaluation of the parameter which indicates the type and intensity of the selection regime acting on a protein-coding gene (Goldman and Yang 1994; Yang et al. 2000; Bielawski 2013).

In contrast to the common practice of inferring substitution-related model parameters, not much work has been devoted to inferring parameters describing indel dynamics, such as the ratio between indel rates and substitution rates and the distribution of indel size. Two notable efforts to infer indel parameters include the lambda.pl script implemented as part of the Dawg package (Cartwright 2005) and an expectation maximization algorithm to infer those parameters from HMMs (Hidden Markov Models) of a pair of sequences (Cartwright 2009). A possible reason for the paucity of analyses for which indel parameters are inferred is that indel dynamics parameters are substantially more challenging to estimate compared with substitution parameters (Cartwright 2005; Fletcher and Yang 2009). Factors contributing to this challenge include the dependency among sites introduced by indel events and the existence of overlapping indels.

Because the indel dynamics parameters are an essential precursor to many phylogenetic procedures, we were motivated to find a way to infer these parameters from input data sets. In this study, we present a novel algorithm to infer the indel to substitution rate ratio, the parameter controlling the distribution of indel length and the root length from an input multiple sequence alignment (MSA) and tree. Our proposed method is general and could be applied to a wide variety of theoretical models. We demonstrate our method by analyzing the indel dynamics in three data sets—from mammals, from the COG (Cluster of Orthologous Groups) database, and from an HIV-1 (Human Immunodeficiency Virus 1) data set—demonstrating the variability of indel dynamics among protein MSAs. We further demonstrate how combining our algorithm with sequence simulators leads to simulated sequences that mimic real sequence data sets in terms of indel prevalence and length. We provide the SPARTA (Searching indel PARameters Trained from Alignment) software implementing our algorithm.

## Materials and Methods

### Indel Dynamics Parameters

In this study, we demonstrate our methodology for the inference of three indel parameters. These parameters are used by the sequence simulation program to generate the evolutionary process along the tree. The first parameter is the indel-to-substitution rate ratio (IR), which controls the proportion of events in the simulation in which an indel is created. The second parameter is the “*a*” parameter of the power law distribution, which controls the distribution of indel length. This distribution describes an inverse relation between the length of an indel and its probability. The third parameter is the length of the sequence at the root of the tree (RL). The simulation is of an evolutionary process along a tree, and this sets the length of the ancestral sequence which is mutated along the branches of the tree (Fletcher and Yang 2009).

### Attributes and Attribute Computation

1. MSA length: The number of columns in the alignment.
2. Total number of gap blocks in the MSA: Gap blocks are one or more consecutive gap characters. This is an estimate of the number of indels per sequence, over the whole MSA.
3. Average gap block length: The total number of gap characters divided by the total number of gap blocks. This is a proxy of average indel length.
4. Minimal length of sequence in the input MSA.
5. Maximal length of sequence in the input MSA.

### Confidence Measure for SPARTA’s Inferred Parameters

The SPARTA methodology can be used to examine the fit between summary statistics (attributes) of the input MSA and those statistics computed from the simulated MSAs under the inferred indel parameters. This is achieved by first computing the Mahalanobis distance between the vector of summary statistics computed from the real data to the multivariate distribution of summary statistics computed from the simulated MSAs. Next, this distance is translated to a *P* value according to the following formula (Clark et al. 1993):

$$p=1-\text{CDF}.\text{CHISQ}(D^{2},v-1)$$

where *D* is the Mahalanobis distance, *v* is the number of summary statistics, and CDF.CHISQ is the chi-squared cumulative distribution function with *v* − 1 degree of freedoms.

A significant *P* value (e.g., smaller than 0.05) means that the vector of summary statistics computed from the real MSA is unlikely to originate from the same multivariate distribution yielding the set of simulated MSAs, suggesting the assumed indel model does not capture some indel aspects reflecting the evolution of the real data analyzed.

### Parameter Configurations

In this study, we examined the inference of the slope of the power law distribution of indel lengths (*a*), the indel-to-substitution rate ratio (IR), and the ancestral sequence length (RL). The following parameter configurations were used in this study:

1. “Basic configuration”: *a* = 1.3, IR = 0.02, RL = 350.
2. Alternative configuration 1: *a* = 1.3, IR = 0.02, RL = 100.
3. Alternative configuration 2: *a* = 1.3, IR = 0.02, RL = 500.
4. Alternative configuration 3: *a* = 1.1, IR = 0.02, RL = 350.
5. Alternative configuration 4: *a* = 1.7, IR = 0.02, RL = 350.
6. Alternative configuration 5: *a* = 1.3, IR = 0.01, RL = 350.
7. Alternative configuration 6: *a* = 1.3, IR = 0.1, RL = 350.

These configurations were simulated using INDELible (Fletcher and Yang 2009). All other INDELible parameters were set to the following: “NUCLEOTIDE 2” model, substitution model: “HKY 2.5,” and maximum indel length: 50. For all simulations, the Azurin tree described below was used.

### Data sets

#### Azurin Data Set

The Azurin protein MSA was downloaded from the HOMESTRAD database (Mizuguchi et al. 1998). This set includes 29 sequences; the MSA length is 215 amino acids. The maximum likelihood phylogenetic tree for this data set was reconstructed using PhyML (Guindon et al. 2010; Criscuolo 2011) with the following parameters: Model of amino acids substitution = WAG (Whelan And Goldman), discrete gamma model, number of categories = 4, tree topology search = best of NNI (Nearest Neighbor Interchange) and SPR (Subtree Pruning Regrafting), and optimizing over all other parameters (“tlr,” proportion of invariant sites estimated from the data). The tree is given in the supplementary information, Supplementary Material online.

#### OrthoMam Data Set

A mammalian collection of orthologous genes was downloaded from the OrthoMam database (Douzery et al. 2014). This collection includeds 498 MSAs which had orthologs across all 40 mammalian sequences. The gene trees provided by OrthoMam were used in all our analyses.

#### COG Data Set

A collection of orthologous genes was downloaded from the COG database (Tatusov et al. 2003). This collection included 100 MSAs. Each of these MSAs contained 40–50 sequences of genes. The maximum likelihood gene tree for each set of orthologs was reconstructed using PhyML with the following parameters: Model of amino acids substitution = WAG, discrete gamma model, number of categories = 4, tree topology search = NNIs, and optimizing over all other parameters (tlr, proportion of invariant sites estimated from the data).

#### HIV-1 Data Set

Sequences of the HIV-1 data set were sampled from the data set used by Penn et al. (2008). Specifically, for this study the amino acids sequences of the genes *env*, *gag*, *nef*, *pol*, *rev*, *tat*, *vif*, *vpr*, and *vpu* from seven subtypes (A, B, C, D, F, G, J) of HIV-1 group M were used. For each of these genes, a data set of 50 sequences was composed by collecting all the sequences of the J, G, F, and D substrains (32 sequences in total) and randomly sampling 6 sequences from each of the A, B, and C substrains. All data sets were aligned with PRANK using the +F argument. Phylogenetic trees were inferred using PhyML with the following parameters: Model of amino acids substitution = WAG, discrete gamma model, number of categories = 4, tree topology search = best of NNI and SPR, and optimizing over all other parameters.

### Inference of Indel Parameters using SPARTA

The SPARTA algorithm was run on all biological protein data sets using the following INDELible (Fletcher and Yang 2009) configuration: “AMINOACID2” model, WAG substitution model and “POW” indel size model, maximum indel length: 50.

### Algorithm Implementation

The algorithm procedure is described in detail in the Results section. The algorithm was implemented in C++ and is freely available at http://www.tau.ac.il/∼talp/supplementary/sparta/sparta.html (last accessed November 19, 2015).

For the simulations that are part of the SPARTA algorithm, we have integrated parts of the INDELible source code. Parameters were optimized using an iterative golden search procedure (Press et al. 2002), starting from the root length. To avoid local maxima, three different root length starting points were used: The length of the shortest sequence, the length of the longest sequence, or the number of columns in the MSA. The two other parameters were always searched starting from IR = 0.075 and *a* parameter = 1.55. For the data analyzed in this work, searches were conducted in the following intervals: IR from 0 to 0.16, *a* from 1 to 2, and RL from 50 to 1,800.

## Results

### The Algorithm

**Fig. 1.—:**
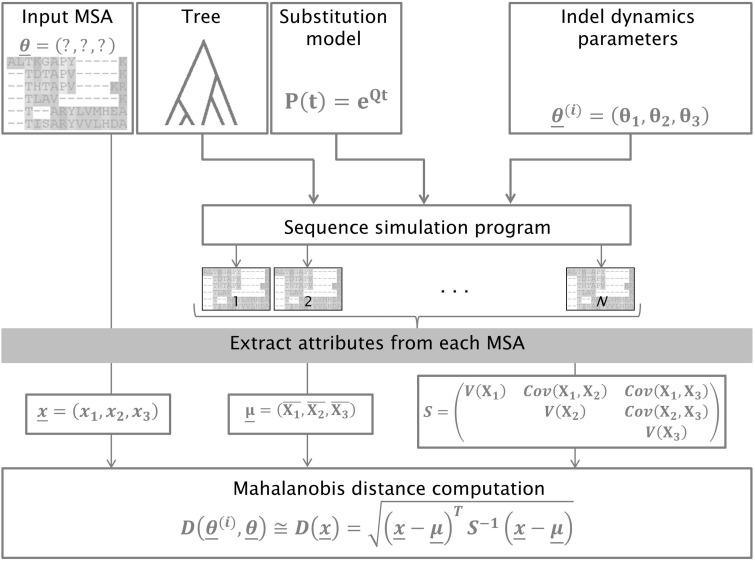
SPARTA methodology uses Mahalanobis distance to measure the fit of proposed parameters to input data. Presented is a single search step, in which the distance between a proposed set of parameters, *Θ*^(*i*)^, and the true unknown parameters *Θ* is computed. Standard hill-climbing heuristics are used to search for a set of parameters that minimizes the distance between simulated data and input data.

Our algorithm takes a given MSA as input and infers three relevant parameters regarding indel dynamics: 1) IR, the indel-to-substitution rate ratio; 2) the slope parameter *a* of the power law distribution, which controls the distribution of the lengths of indels; and 3) RL, the length of the sequence at the root of the phylogeny (for more details, see Materials and Methods). Our proposed methodology uses a simulation-based approach to search over the space of parameters for the ones that best fit the input MSA. In each search step, a specific set of input parameters is estimated. This set is refined (using standard hill-climbing heuristics) until an optimal set of parameters is inferred. The fit of each set of parameters to the input MSA is computed as a measure of the distance between the input MSA and a set of MSAs produced by simulating sequences under this set of parameters. In order to calculate this distance, we compute a vector of attributes for the input MSA as well as for each of the simulated MSAs produced under the set of parameters of the current step. These attributes are summary statistics computed from each MSA (e.g., the average gap block length and the number of gap blocks, see Materials and Methods). We next compute the Mahalanobis distance (Mahalanobis 1936) between the attribute vector of the input MSA and the distribution of attribute vectors computed for each of the *N* simulated MSAs in the current step:

$$\text{D}\left(\underline{x}\right)=\sqrt{{\left(\underline{x}-\underline{\mu }\right)}^{\text{T}}{\text{S}}^{-1}\left(\underline{x}-\underline{\mu }\right)}$$

where $\underline{x}$ is the vector of attributes computed for the input MSA, $\underline{\mu }$ is the vector of attribute means for the set of simulated MSAs, and S is the covariance matrix for the set of simulated MSAs. Our working hypothesis is that this distance is a good estimate of the difference between the current search step parameters and the parameters underlying the input MSA, that is, a large $\text{D}\left(\underline{x}\right)$ value indicates that the current set of parameters is unlikely to generate MSAs with the same characteristics as the input MSA. Our parameter search procedure, SPARTA, is described schematically in figure 1. An example of the dependency of the Mahalanobis distance surface for a grid of parameter combination is presented in supplementary figure S1, Supplementary Material online, which shows that indeed the Mahalanobis distance increases as the parameter values are further away from the true set of parameters.

### Tuning the Methodology and Run-Time Analysis

**Fig. 2.—:**
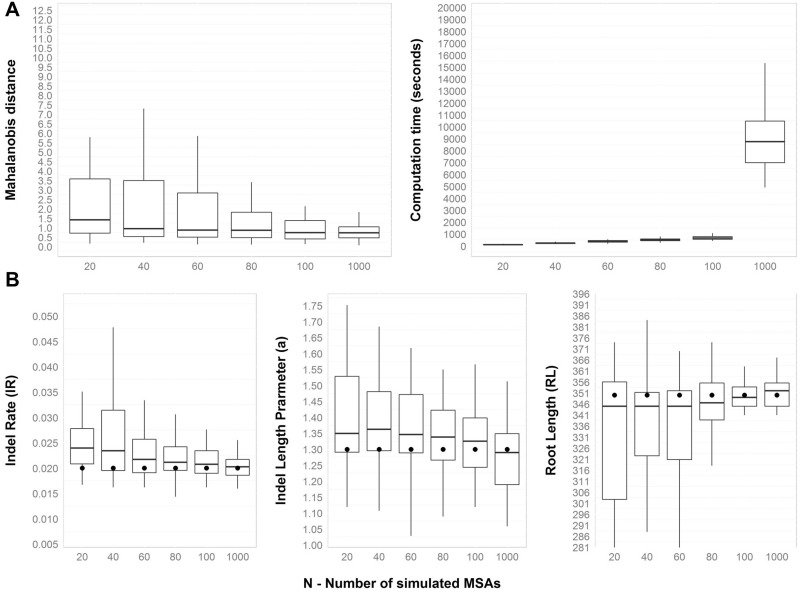
Inference accuracy is positively correlated with the number of simulated MSAs (*N*) used in each search step. Fifty “real” MSAs were simulated using the basic parameter configuration (see Materials and Methods). The parameters of each of these MSAs were then searched for, with different values of *N*. Panel *A* depicts the Mahalanobis distance and the computation time as a function of *N* and panel *B* shows how each of the inferred parameters depends on *N*. The real parameter values are marked as bold points.

We first set out to fine tune our methodology, which depends on the number *N* of simulated MSAs. To this end, we simulated a set of MSAs with known parameters under a specific tree topology and branch lengths and tested the ability of our methodology to accurately infer parameters. Specifically, the tree topology and branch lengths were chosen to reflect a biological data set (see Materials and Methods—the Azurin data set), and different configuration sets were used to model indel dynamics. Using the basic configuration set (see Materials and Methods), we simulated 50 MSAs using INDELible (Fletcher and Yang 2009). As we show below, this parameter configuration is well within the range of biologically plausible parameters. SPARTA then estimated the indel parameters of these 50 simulations. Figure 2*A* summarizes the dependence of the Mahalanobis distance on *N* (and provides information regarding run times), while figure 2*B* shows the distance between each “true” parameter and the inferred one, as a function of *N*. As expected, the accuracy of the parameter search procedure increases with *N*. We chose *N* = 100 for further analyses as this value offers a good compromise between accuracy and computation time. For *N* = 100, the average inferred parameters and one standard error were indel rate = 0.023 ± 0.006, *a* parameter = 1.324 ± 0.116, and root length = 341.94 ± 24. 764. These values well fit the real parameters: 0.02, 1.3, and 350.

### Accuracy Evaluation

We next evaluated the performance of our methodology as a function of the parameters used to simulate the true MSA. We aimed to determine the accuracy as a function of the above presented three parameters: Low versus high number of indel events, shorter versus longer indels, and different root lengths. To this end, we tested the basic parameter configuration as well as six alternative parameter configurations, each of which differs from the basic parameter set by the value of one parameter (see Materials and Methods). These configurations were chosen to represent a wide range of evolutionary scenarios. The results of these analyses are presented in table 1. As can be seen, in all parameter configurations, the real parameter values fall within one standard error from the inferred parameter value. Thus, the algorithm is able to reconstruct a broad spectrum of parameter values. The results suggest an increased accuracy for high values of indel rate which may be explained by the presence of more indel events from which reliable estimates may be obtained.

**Table 1:** Method Accuracy for Different Parameter Combinations

Various indel rates			
RL = 350 *a* = 1.3	IR = 0.01	IR = 0.02	IR = 0.1
Inferred IR	0.016 ± 0.017	0.023 ± 0.006	0.102 ± 0.013
Inferred *a*	1.379 ± 0.157	1.324 ± 0.116	1.297 ± 0.061
Inferred RL	337.1 ± 37.69	341.94 ± 24.76	344.74 ± 23.18
Various slope parameter values			
IR = 0.02 RL = 350	*a* = 1.1	*a* = 1.3	*a* = 1.7
Inferred IR	0.029 ± 0.018	0.023 ± 0.006	0.021 ± 0.003
Inferred *a*	1.261 ± 0.151	1.324 ± 0.116	1.686 ± 0.16
Inferred RL	319.58 ± 40.19	341.94 ± 24.76	350.36 ± 5.34
Various root lengths			
IR = 0.02 *a* = 1.3	RL = 100	RL = 350	RL = 500
Inferred IR	0.021 ± 0.006	0.023 ± 0.006	0.023 ± 0.011
Inferred *a*	1.273 ± 0.157	1.324 ± 0.116	1.379 ± 0.093
Inferred RL	99.14 ± 7.84	341.94 ± 24.76	489.72 ± 25.42

Note.—Data sets were simulated according to seven alternative parameter configurations. Fifty MSAs were simulated by each configuration and were given as input to SPARTA in order to evaluate its ability to accurately infer the parameter values. Each value is the average inferred parameter and one standard error.

### Comparison with Dawg Parameter Inference

**Fig. 3.—:**
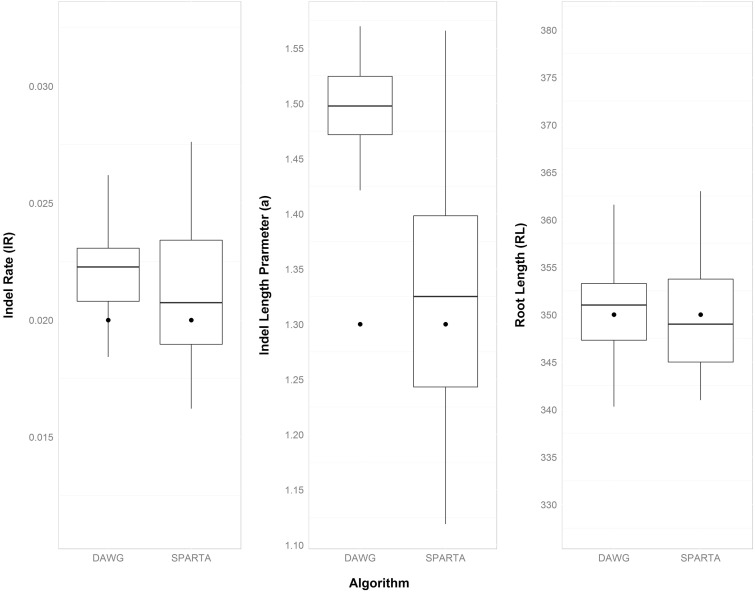
SPARTA’s inference is better than lambda.pl’s. Fifty “real” MSAs simulated using the basic parameter configuration were given as input to SPARTA as well as to Dawg’s lambda.pl script. The real parameter values are marked as bold points.

A previous effort to infer indel parameters is implemented as a Perl script lambda.pl which is part of the Dawg package (Cartwright 2005). We compared the performance of parameter estimation by SPARTA with that by the Dawg package using simulations. The accuracy performance on the basic parameter configuration is shown in figure 3. As can be seen, for this set of parameters, lambda.pl overestimates the *a* parameter (i.e., shorter indels) and is thus less accurate than SPARTA. The difference in accuracy between the method for this parameter is statistically significant (comparing the squared errors between the true parameter values and the inferred ones using Wilcoxon paired test; *P* < 8.44 × 10^−8^). No statistically significant difference between the two methods was found for the estimation of the indel rate parameter. SPARTA was slightly less accurate for the inference of the root length parameter (*P* < 2.7 × 10^−3^). We repeated this comparison for all six alternative parameter sets (see Materials and Methods). The results (supplementary fig. S2, Supplementary Material online) suggest that lambda.pl tends to overestimate the *a* parameter for many of the examined parameter sets. Furthermore, we counted the cases in which the real parameter values fell within a single standard error from the average inferred parameters. While for SPARTA this was the case for all sets of parameters examined, Dawg managed to correctly infer only a single complete set. These results suggest that SPARTA is a valuable alternative to Dawg for accurate inference of indel parameters.

### Inference under Different Alignment Programs

**Fig. 4.—:**
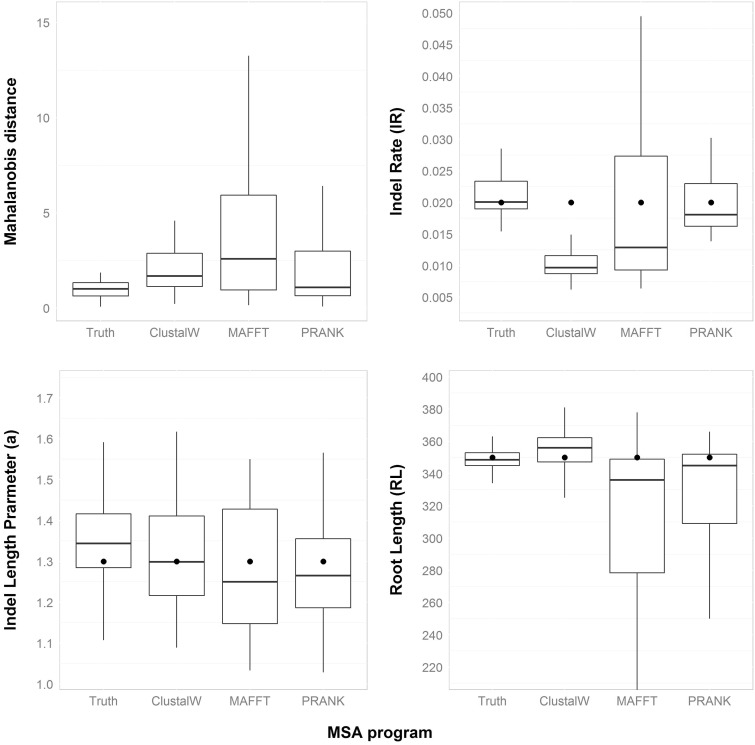
SPARTA’s inference is robust to biases introduced by MSA programs. Fifty sequence data sets obtained using the basic parameter configuration were aligned by either ClustalW, MAFFT, or PRANK. The MSAs computed by each alignment program were given as input to SPARTA. The real parameter values are marked as bold points. As reference, we also present the inferred values using the “true” MSAs generated by INDELible.

In all the above described analyses, the attributes used for the inference of indel dynamics parameters were extracted from the true MSA. In practice, however, the true MSA is not available and an MSA has to be reconstructed from the sequence data by an alignment program. Our next goal was to study the impact of commonly used alignment programs on the performance of SPARTA. To this end, we examined ClustalW V1.8 (Thompson et al. 1994; Higgins et al. 1996), MAFFT V7 (Katoh and Standley 2013), and PRANK V140603 (Loytynoja and Goldman 2008; Loytynoja 2014). We simulated sequence data sets using INDELible under the basic parameter configuration. The simulated unaligned sequences were given to each of the alignment programs to compute MSAs. Those MSAs were next used as input MSAs for the algorithm and the accuracy of the algorithm was evaluated. As can be seen in Figure 4, for all three alignment programs considered, the inference of the *a* parameter and the root length were relatively accurate despite the fact that the MSA is inferred rather than known. Regarding the estimation of the indel rate, both MAFFT and PRANK allow for accurate estimation of this parameter. However, this parameter is substantially underestimated when the MSA is reconstructed using ClustalW. This is in accordance with previous reports suggesting that ClustalW tends to overalign sequences (Loytynoja and Goldman 2008; Privman et al. 2012). We repeated this analysis for all six alternative parameter configurations (see Materials and Methods). The results (supplementary fig. S3, Supplementary Material online) further support our observation for the basic parameter set, that is, the indel rate parameter in ClustalW MSAs tends to be underestimated. Moreover, following alignment by all MSA programs, the indel rate parameter was underestimated under the high indel rate configuration, with alignments by PRANK and MAFFT yielding closer estimates to the true value compared with ClustalW.

### Simulating Data with SPARTA Parameter Estimates

**Fig. 5.—:**
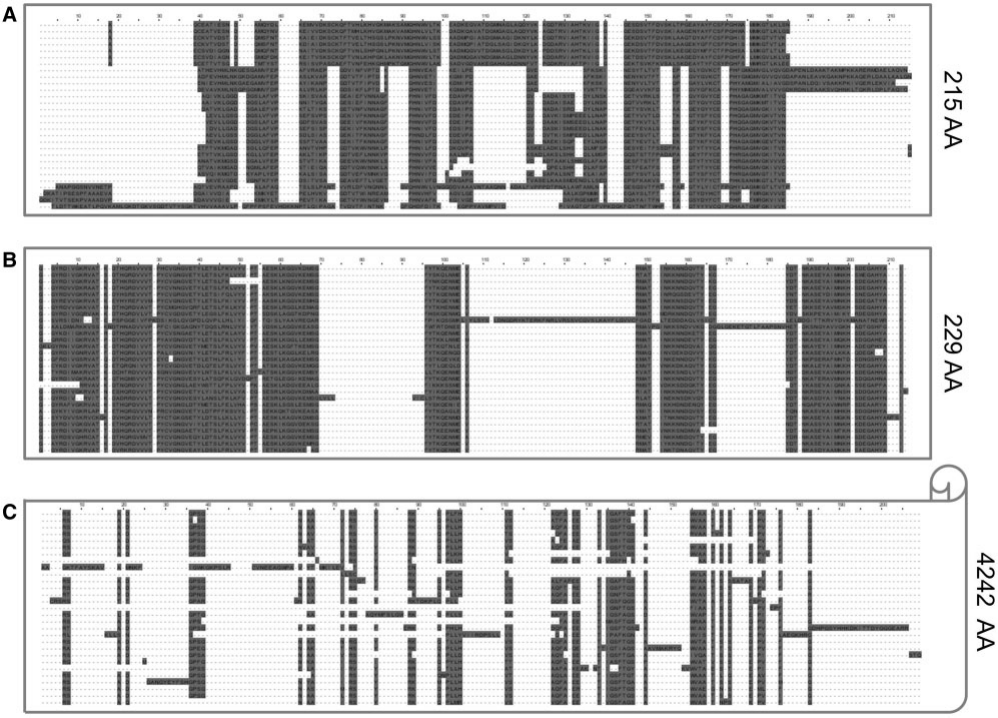
SPARTA can be used to simulate MSAs similar to a target MSA. The plot depicts three MSAs. The real Azurin MSA (panel A), a simulated MSA using the parameters the algorithm inferred for the Azurin MSA (IR = 0.0135, *a* = 1.325, RL = 119; panel *B*) and a simulated MSA using INDELible’s default parameters (as described in the Materials and Methods section) (panel *C*). As the MSA simulated based on the default parameters is 4,242 amino acids long, only the first 200 columns are presented in the plot.

Sequence simulators are used for a wide variety of phylogenetic analyses (Fletcher and Yang 2010; Bay and Bielawski 2011, 2013; Gaston et al. 2011; Izquierdo-Carrasco et al. 2011; Wu and Susko 2011; Blackburne and Whelan 2012; Jordan and Goldman 2012; Kuck et al. 2012; Loytynoja et al. 2012; Privman et al. 2012; Thi Nguyen et al. 2012; Wang et al. 2013; Redelings 2014; Spielman et al. 2014). For each sequence simulation, specific models are assumed, and investigators must provide the model parameters. Given a specific indel model used by the simulator, the task of selecting its parameters so that the simulator could produce MSAs similar in their indel characteristics to the studied MSA is not a trivial one. Notably, the selection of indel parameters for a given MSA motivated the inclusion of lambda.pl as part of the Dawg simulation package. To demonstrate the utility of SPARTA to infer such parameters, we aimed to simulate MSAs that resemble the indel characteristics of the Azurin protein MSA (see Materials and Methods, Azurin data set,) shown in figure 5*A*. A typical MSA simulated based on the parameters inferred by our methodology is presented in figure 5*B*. As can be seen, the real MSA and the simulated MSA are similar with respect to their total length and the number and size of indels. In contrast, a typical MSA simulated with the default INDELible parameters (fig. 5*C*) is characterized by shorter indels and a much longer alignment length compared with the true MSA. In both simulations, the Azurin tree topology and branch lengths were used and the same substitution model was assumed.

### Indel Dynamics in Biological Data Sets

**Fig. 6.—:**
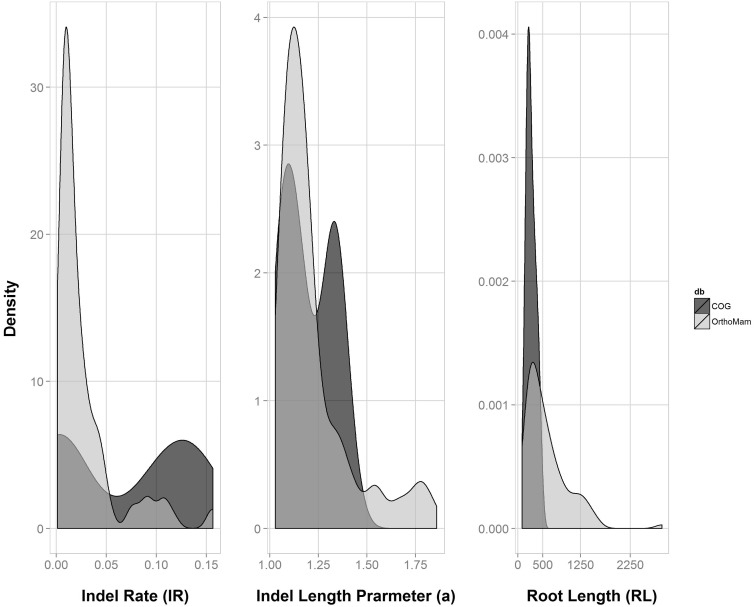
Distribution of parameter values in real data sets. The algorithm was run on 498 mammalian MSAs obtained from the OrthoMam database as well as 100 COG MSAs. The panels depict the distribution of the inferred parameter values in cases where the *P* value was not significant (*P* > 0.05; 104 OrthoMam genes and 28 COG genes).

Having established that our method can accurately infer indel dynamics parameters, we next applied our method to study real biological data sets. We examined 498 mammalian MSAs obtained from the OrthoMam V8 database (Douzery et al. 2014) and 100 MSAs obtained from the COG database (Tatusov et al. 2003). We ran the search algorithm on each such MSA to infer its parameters (fig. 6). Our results show that indel dynamics differ between mammals and the COG MSAs that include a relatively diverse set of (mostly microbial) organisms. COG MSAs are characterized by a narrow distribution of short root lengths. Furthermore, the indel rate is higher in COG MSAs compared with mammalian MSAs, indicating that indel dynamics vary among different clades. Notably, when analyzing such real biological data sets, additional sources of bias regarding MSA inaccuracies exist, for example, the inclusion of paralogs instead of orthologs, the inclusion of only partial sequences, or biases due to filtering of specific sequences and positions. Nevertheless, the above results demonstrate the utility of our method to estimate indel parameter distributions for various phylogenetic groups.

**Fig. 7.—:**
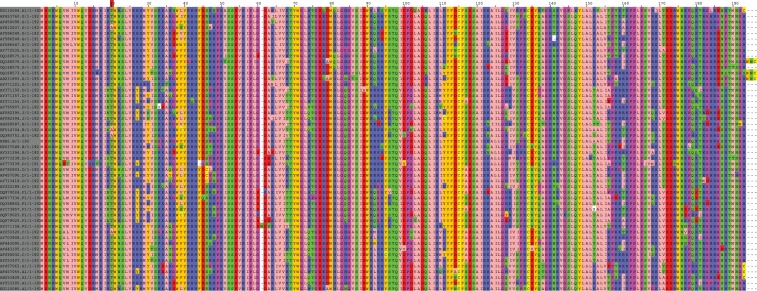
PRANK MSA of the *vif* protein across 50 HIV-1 samples.

In addition, we inferred the indel dynamics parameters for nine HIV-1 coding genes. As can be seen in table 2, there is at least an order of magnitude variation in the inferred indel rate parameter, ranging between 0.0003 and 0.0195 indels to substitutions. These values are lower than those inferred for the OrthoMam and COG data sets, suggesting that indels are less common compared with substitutions for HIV-1 than for mammals and bacteria. Furthermore, experimental data suggest that indel mutations comprise 3–6% of the total cases of mutations (Abram et al. 2010), which is approximately the values inferred for seven out of the nine HIV-1 genes we analyzed. For two HIV-1 genes, *vif* and *vpr*, a much lower indel rate parameter value was inferred (table 2). This result is in line with the low number of indels compared with substitutions observed in the MSAs of these genes (the alignment of *vif* is shown in fig. 7). The low indel rate ratio of these genes compared with the other HIV-1 protein-coding genes suggests these two genes are either subject to a different mutation regime or to a stronger purifying selection against the introduction of indels.

**Table 2:** Indel Dynamics Parameters Inferred using SPARTA from Nine HIV-1 Protein MSAs

Gene	IR	*a*	RL	*P* value
*env*	0.1322	1.55	728	1.00 × 10^−14^
*gag*	0.0195	1.8441	494	0.648083
*nef*	0.0195	1.1375	181	0.164597
*pol*	0.0872	1.6744	881	1.00 × 10^−14^
*rev*	0.009	1.6586	118	0.997467
*tat*	0.0095	1.5756	97	0.997941
*vif*	0.0035	1.9014	192	0.989768
*vpr*	0.0004	1.55	96	0.999988
*vpu*	0.0039	1.2163	451	1.00 × 10^−14^

Note.—Each MSA was composed of 50 orthologs.

## Discussion

In this study, we presented a novel method to infer indel dynamics parameters from an input MSA by extracting summary statistics. Specifically, we demonstrated the ability of the algorithm to recover three parameters. However, our method is general and is not limited to a specific theoretical model of indels and could be, with minor adjustments, applied to study the parameters of other theoretical models describing indel dynamics. As a case in point, it can be used to study other indel size distributions, such as the Negative Binomial distribution (Popescu 2003; Fletcher and Yang 2009).

The last years have seen a steady progress toward advanced Bayesian approaches aimed at reconstructing trees and MSAs simultaneously (Thorne et al. 1992; Lunter et al. 2005; Redelings and Suchard 2005; Bouchard-Cote and Jordan 2013; Herman et al. 2014). One of the strengths of these approaches is that they integrate over uncertainty in model parameters, including those relevant to indel dynamics. Thus, such methodologies can, in principle, provide posterior estimates for indel dynamics parameters. Unfortunately, such methods are generally computationally intensive and hence these approaches cannot be easily applied to data sets containing hundreds or thousands of taxa.

Similar to these Bayesian methodologies, the SMUVE approach (Cartwright 2009; software available at https://github.com/reedacartwright/emdel) presents a likelihood-based model to infer indel dynamics parameters, using pairwise HMMs. Although this approach is clearly more powerful than previously developed methods (discussed in Cartwright 2009), it is limited to the inference from pairwise alignments only.

In this study, we compared our approach with a methodology for the inference of indel parameters implemented in lambda.pl, part of the Dawg package. Lambda.pl first estimates the number of unique gap characters, and from which, assuming a Poisson distribution, it infers the ratio between indel and substitution events. Furthermore, from the estimated size frequencies of the unique gaps, it infers the parameters of the indel size distribution. The inaccuracies we observed when testing the performance of lambda.pl may stem from errors in counting unique indels (e.g., due to overlapping indel events or parallel indel formation). Notably, SPARTA as opposed to lambda.pl does not require a rooted tree, which is often unavailable (e.g., when gene trees are analyzed).

Furthermore, our proposed methodology opens the way to account for indels in simulation studies by obtaining indel dynamics parameters which were inferred from biological data sets and subsequently generating sequences that evolved under these parameters. Our methodology can further be used in tests based on parametric bootstrap. Although previously developed parametric bootstrap methodologies have simulated sequences without indels (Bull et al. 1993; Swofford et al. 1996), it is now possible to simulate alignments with indel parameters that have been estimated from the real MSA. Parametric bootstrapping with indels may be very important in cases where alignment uncertainty may affect downstream analyses, for example, when inferring positive selection (Jordan and Goldman 2012; Privman et al. 2012; Blackburne and Whelan 2013; Redelings 2014; Spielman et al. 2014) and testing if two or more trees are equally supported by the data (Levy Karin et al. 2014). Because in many cases it is unknown if indels in the alignment affect downstream analyses, we suggest that whenever a parametric bootstrap approach is utilized, indels should be accounted for by simulating with indel parameters that are estimated from real data sets.

In addition to utilizing SPARTA together with a sequence simulator for simulating data sets that resemble an input MSA (as shown in fig. 5), our method can also be utilized to detect indel dynamics parameters for specific genes or lineages. We have demonstrated these possible uses by comparing indel rates among mammalian, bacterial, and viral sequences (fig. 6) and among HIV-1 genes (fig. 7). Differences in indel dynamics may reflect changes in either the mutation process or in the selection regime. For example, a higher indel rate in a given gene may stem from relaxation in purifying selection or, in rare cases, from positive selection.

We applied SPARTA to a large number of biological data sets and tested whether the real summary statistics were consistent with the distribution of summary statistics generated by simulations using the inferred indel parameters. A large fraction of data sets were significantly different (α = 5%) than their associated simulations (394 of 498 Orthomam data sets, 72 of 100 COG data sets, and 3 of 9 HIV-1 MSAs). This suggests that further improvements in indel modeling are needed. For example, currently it is assumed that the indel parameters are shared across all positions, while it is plausible that indels are more likely in some regions than others. Similarly, indel dynamics may vary across tree lineages. These aspects await further research.

It should be noted that the methodology presented here infers indel dynamics from input MSAs that were themselves generated by MSA programs that assume specific indel parameters and are subjected to various biases (Lunter et al. 2008). One could claim that such an approach may be biased toward inferring the parameters used to reconstruct the input MSAs. Furthermore, it is well established that the “optimal” MSA obtained by each alignment program reflects only one possible path out of many equally likely paths and many more suboptimal solutions (Landan and Graur 2007). One possible improvement of the SPARTA methodology would be to account for MSA uncertainty by averaging the parameter estimations over a large sample of plausible MSAs. Such alternative MSAs can be obtained using the GUIDANCE methodology (Penn et al. 2010; Sela et al. 2015) or statistical MSA methodologies (Hein et al. 2000; Herman et al. 2015). Although it would be computationally expensive, biases introduced by MSA programs can be incorporated into SPARTA by realigning simulated MSAs using the same aligner as the original data set and comparing the results with the input MSA.

Our methodology currently estimates the best set of indel dynamics parameters that match a specific input MSA. In the future, the method can be extended to compute not only optimal parameters, but rather a distribution of plausible parameters for each input alignment. This can be achieved using methodologies such as approximate Bayesian computation (Marjoram et al. 2003), which are already extensively used in population genetics studies (Blum and Jakobsson 2011; Shafer et al. 2015).

## Supplementary Material

Supplementary information and figures S1–S3 are available at *Genome Biology and Evolution* online (http://www.gbe.oxfordjournals.org/).

Supplementary Data
