# Supplementary material for: Inferring Indel Parameters using a Simulation-based Approach
Source: Genome Biol Evol. 2015 Nov 3;7(12):3226–38. doi: 10.1093/gbe/evv212 (PMC4700945; doi:10.1093/gbe/evv212)
Supplement: Supplementary Data [file supp_7_12_3226__index.html]

Inferring Indel Parameters using a Simulation-based Approach — Supplementary Data 

# Inferring Indel Parameters using a Simulation-based Approach

## Supplementary Data

files

- Supplementary Data - pdf file
