# Supplementary material for: Inferring Indel Parameters using a Simulation-based Approach
Source: Genome Biol Evol. 2015 Nov 3;7(12):3226–38. doi: 10.1093/gbe/evv212 (PMC4700945; doi:10.1093/gbe/evv212)
Supplement: Supplementary Data [file supp_evv212_SuppMaterial_sparta.pdf]

Supplementary information for: Inferring indel parameters using a simulation-based approach

Eli Levy Karin*[1], Avigayel Rabin*[1], Haim Ashkenazy[1], Dafna Shkedy[1], Oren Avram[1,2], Reed A. Cartwright[3,4] and Tal Pupko[§1]

[1] Department of Cell Research and Immunology, George S. Wise Faculty of Life Sciences, Tel-Aviv University, Tel-Aviv 69978, Israel

[2] The Blavatnik School of Computer Science, Tel-Aviv University, Tel-Aviv 69978, Israel

[3] The Biodesign Institute, Arizona State University, Tempe, AZ, USA

[4] School of Life Sciences, Arizona State University, Tempe, AZ, USA

*Equal contribution

[§]Corresponding author

Tal Pupko, Tel: 972-3-6407693; Fax: 972-3-6422046

E-mail: talp@post.tau.ac.il

The Azurin phylogenetic tree

(2azaa:0.06641051,1dz0a:0.07290840,(((1joi:0.07049126,1nwpa:0.08388152)0.996000:0.18
233892,(1jzga:0.16919013,1rkra:0.14102060)0.319000:0.07245806)0.750000:0.06007521,(1
cuoa:0.16317837,((1qhqa:0.42107153,1gy1a:2.13155402)0.631000:0.14145797,(1kdj:0.738
28629,((1iuz:0.07061881,7pcy:0.08099794)0.936000:0.15562908,((2plt:0.14249476,(2b3ia:0
.45655793,(((1bawa:0.16998276,1nin:0.35656989)0.726000:0.12232571,((2raca:0.31062429
,1id2a:0.19115406)0.957000:0.69045710,(1pmy:0.52396051,(1bqk:0.09688485,(1adwa:0.27
091378,1paz:0.27479179)0.820000:0.07646418)0.785000:0.18447640)0.991000:0.8317950
3)0.731000:0.39896490)0.339000:0.09829161,(1m9wa:0.30473764,1bxva:0.24961059)0.035
000:0.06117362)0.622000:0.10064130)0.998000:0.37727856)0.458000:0.12870291,(1plb:0.
09351592,(1byoa:0.10581124,(9pcy:0.11845340,(1ag6:0.05796853,1plc:0.18318300)0.0000
00:0.02426169)0.868000:0.06565479)0.919000:0.11207196)0.967000:0.23647586)0.751000
:0.04458255)0.783000:0.23455363)0.989000:0.89059263)0.983000:0.90598315)0.937000:0.
40986130)0.967000:0.16070052);

Figure Legends

Figure S1 The dependence of the Mahalanobis distance as a function of different parameter combinations (the IR and the '*a*' parameter). The RL value was 350 basepairs and was given to the algorithm. The real parameters were IR = 0.02 and '*a*' parameter = 1.3. It can be seen the Mahalanobis distance increases as the tested parameters are further away from the real ones. The MSA used as input was simulated under the "basic parameter configuration" (see Materials and Methods).

Figure S2 Parameters inference by SPARTA and by Dawg. Fifty real MSAs obtained using each of the six alternative parameter combinations (see Materials and Methods) were given as input to SPARTA as well as to Dawg lambda.pl script. The real parameter values are marked as bold points.

Figure S3 Inference accuracy using three different alignment programs. Fifty sequence datasets obtained using each of the six alternative parameter combinations (see Material and Methods) were aligned by either ClustalW, MAFFT or PRANK. The MSAs computed by each alignment program were given as input to SPARTA. The real parameter values are marked as bold points. As reference, we also present the inferred values using INDELible's true MSAs.

Figures

Figure S1

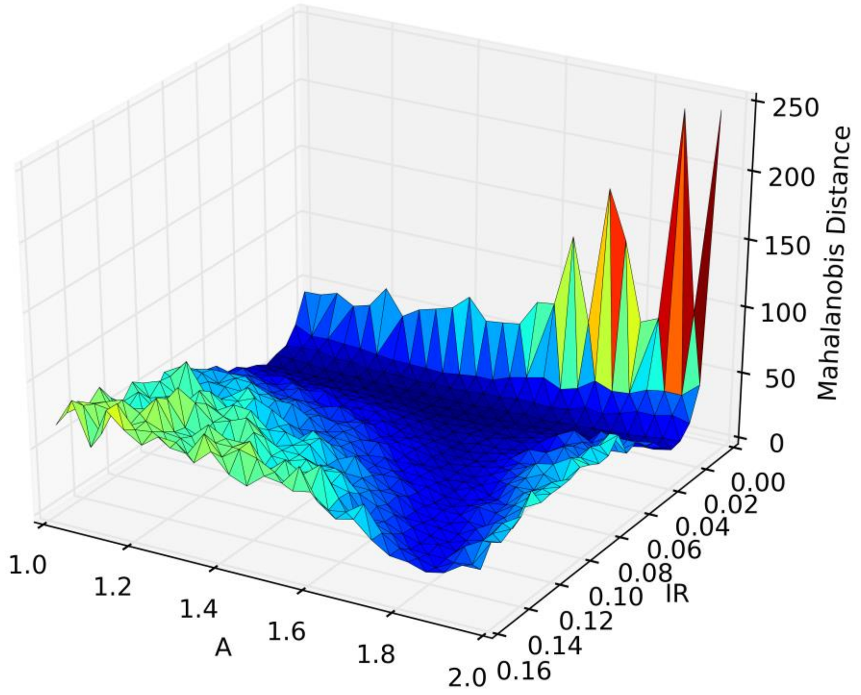

Figure S2

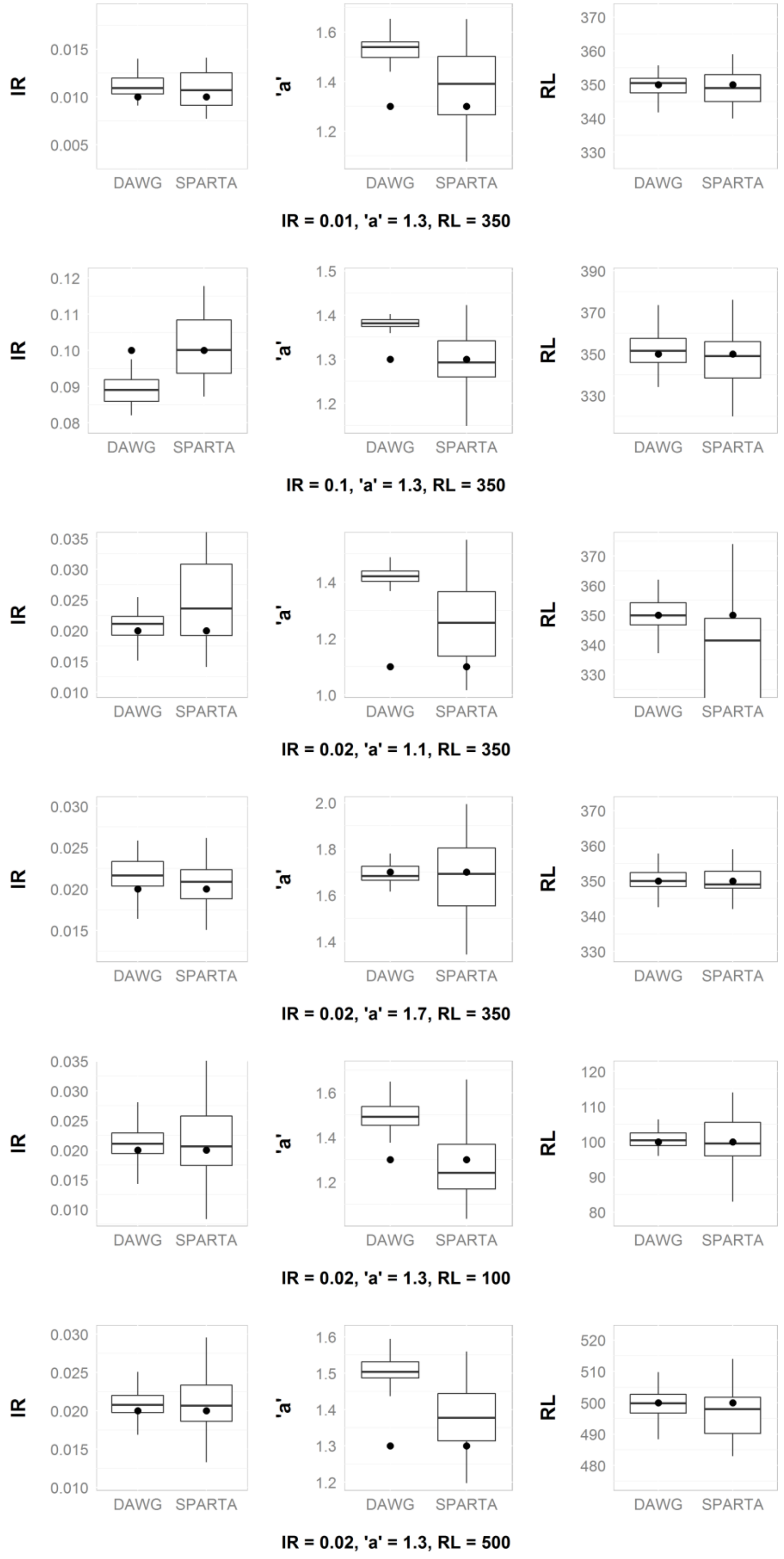
IR
'a'
RL
DAWG
SPARTA
IR = 0.01, 'a' = 1.3, RL = 350
IR = 0.1, 'a' = 1.3, RL = 350
IR = 0.02, 'a' = 1.1, RL = 350
IR = 0.02, 'a' = 1.7, RL = 350
IR = 0.02, 'a' = 1.3, RL = 100
IR = 0.02, 'a' = 1.3, RL = 500

Figure S3

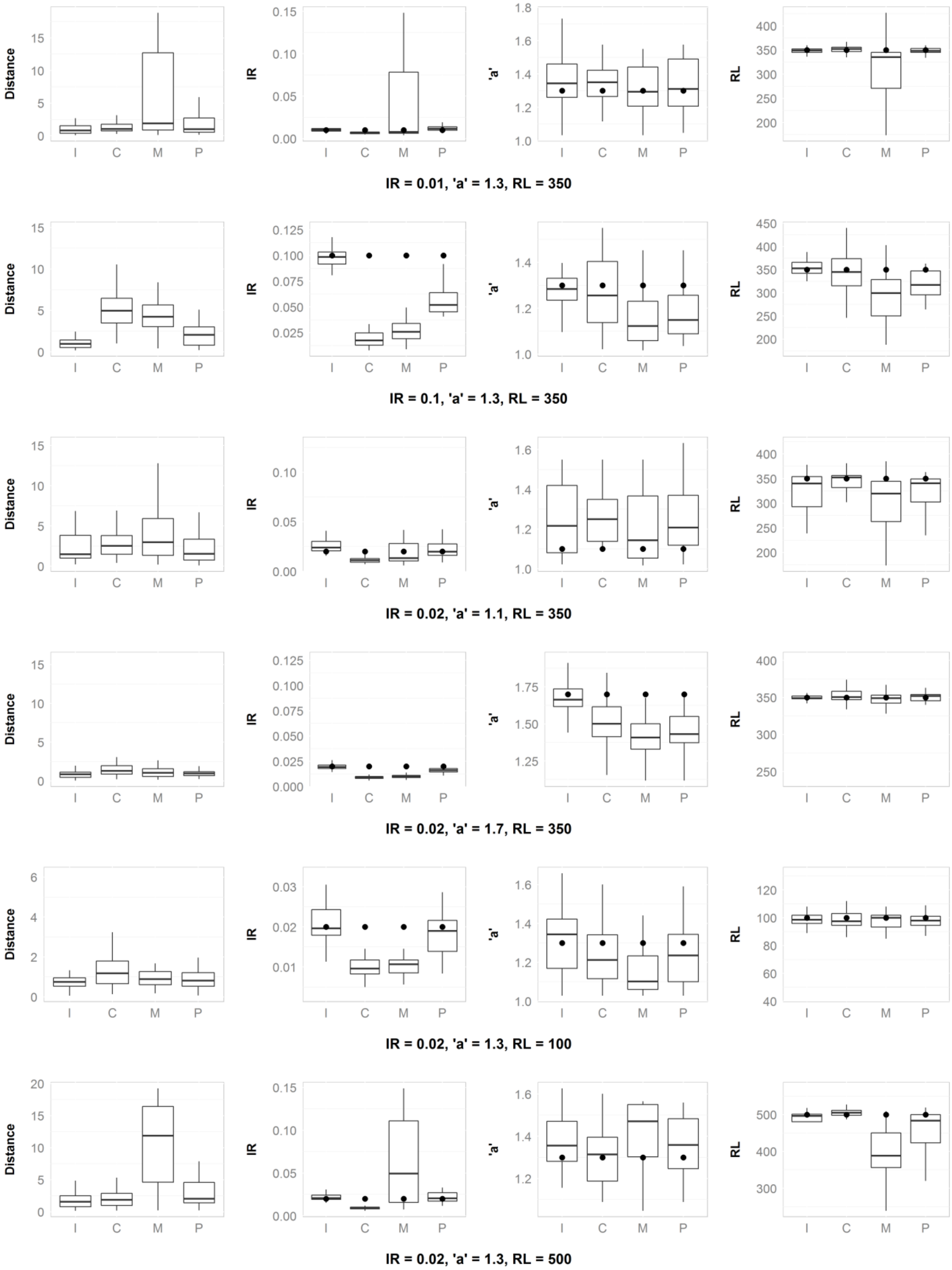
Distance
IR
'a'
RL
I C M P
IR = 0.01, 'a' = 1.3, RL = 350
IR = 0.1, 'a' = 1.3, RL = 350
IR = 0.02, 'a' = 1.1, RL = 350
IR = 0.02, 'a' = 1.7, RL = 350
IR = 0.02, 'a' = 1.3, RL = 100
IR = 0.02, 'a' = 1.3, RL = 500
